# Editorial: Shedding Light on the Nervous System: Progress in Neurophotonics Research

**DOI:** 10.3389/fncir.2022.901376

**Published:** 2022-05-17

**Authors:** Edward S. Ruthazer, Jean-Claude Béïque, Yves De Koninck

**Affiliations:** ^1^Department of Neurology and Neurosurgery, Montreal Neurological Institute-Hospital, McGill University, Montreal, QC, Canada; ^2^Brain and Mind Research Institute, University of Ottawa, Ottawa, ON, Canada; ^3^Department of Psychiatry and Neuroscience, Institut Universitaire en santé mentale de Québec, Université Laval, Quebec City, QC, Canada

**Keywords:** optogenetics, calcium imaging, fluorescence, microscopy, methods, review

The goal of understanding the brain is more than just a scientific endeavor. It is an existential aspect of human nature to want to understand what underlies our own consciousness and thought processes. But the awesome complexity and interconnectivity of the brain pose many challenges to our traditional reductionist approaches to understanding biological phenomena. While nobody actually believes that individual neurons have ideas or that synapses have memories, our best hope for understanding the mechanisms that endow this remarkable organ with the ability to write novels and cure diseases is to study the behavior and interactions of those unitary elements from which the brain is assembled. Addressing this task with the required spatial and temporal scale poses a formidable experimental challenge. True scientific progress and opportunities often occur when distinct fields of study collide; the merging of traditional neuroscience approaches with the ability to control light in space and time certainly provides an explicit and spectacular example. Still in its infancy, the expanding field of neurophotonics has already singularly propelled brain research forward. Broadly defined, neurophotonics is the use of light to measure or manipulate the form and function of individual or ensembles of brain cells, from neurons to glia, even extending to neurovascular coupling. It is in this ability to individually and non-invasively read and control the activity and signaling of multiple brain cells and even synapses, where the neurophotonic revolution has truly shone.

Arguably it was the timely convergence of few key optical and genetic breakthroughs in the past quarter-century that helped reveal the awesome potential of optical approaches to studying the brain. The original cloning, optimization and expression of Enhanced Green Fluorescent Protein (EGFP) derived from bioluminescent *Aequoria victoria* jellyfish opened the door to a broad palette of genetically-encoded fluorescent proteins with diverse spectral and optical properties (Tsien, 1998; Shaner et al., 2005). In addition to a seemingly limitless number of fusion proteins used to study subcellular protein distribution and interactions, perhaps the most functionally significant application of EGFP derivatives has been the development of genetically-encoded calcium indicators (GECIs), like GCaMP and R-GECO, that permit single cell calcium transients to be monitored throughout a neuronal network (Chen et al., 2013; Enterina et al., 2015). The ability to simultaneously detect neuronal and glial activation in hundreds or even thousands of cells non-invasively has been a game-changer for understanding how networks of brain cells function and dynamically interact both *in vitro* and *in vivo*.

This approach, monitoring activity across cells in the brain, was further enhanced by the discovery that the light-gated ion channel called channelrhodopsin-2, derived from the phototactic green alga *Chlamydomonas reinhardtii*, could be used to optically drive action potential firing when expressed in neurons (Nagel et al., 2003; Boyden et al., 2005). A bevy of additional light-gated channels and transporters were identified and engineered to permit precise activation and suppression of neural activity using light in genetically defined populations of brain cells (Kim et al., 2017). Thus, with the emergence of these families of genetically-encoded tools to monitor and to alter neural activity, the field of optogenetics was born. The neuroscientist's optical armamentarium was no longer restricted to observing neural activity, but now squarely included the ability to perturb it with unprecedented ease and precision.

The last element that has truly brought the applications for these powerful tools into clear focus is the microscope itself. While there is no doubt that optogenetics has been exploited brilliantly using conventional microscopy of cultured cells, as well as fiber fluorimetry and wearable 1p fluorescence miniature microscopes *in vivo*, it was the contemporary development of non-linear optical fluorescence microscopy, especially two-photon laser-scanning microscopy (2PLSM), that permitted the full potential of EGFP and GECIs to be applied in the mammalian brain. By restricting fluorescence excitation exclusively to a focal plane, 2PLSM allows deep tissue single-plane imaging as well as full 3D imaging of labeled cells without the severe image degradation caused by out-of-focus fluorescence excitation that plagues conventional microscopy in scattering tissue like brain (Svoboda and Yasuda, 2006). An alternative approach to solving the problem of image haze in more optically accessible translucent organisms like zebrafish larvae (or optically cleared fixed tissue samples) is selective plane illumination microscopy (SPIM), sometimes called light-sheet microscopy, that allows the entire volume of a zebrafish brain to be imaged by repeatedly scanning a thin plane of excitation light up and down through the fish at high speed while acquiring diffraction-limited images with a sensitive digital camera (Huisken and Stainier, 2009). The growing array of sensors, into neurotransmitter and enzymatic sensors, and emergent means to target them to specific cell compartments, coupled with fiber-optic probes to deliver and collect light deep in the brain, open unprecedent capabilities to link intricate cell signaling to behavior, ranging from single-cell photometry to measurements from large ensembles. At the other extreme of imaging approaches, is superresolution nanoscopy, which includes single-molecule localization techniques like direct stochastic optical reconstruction microscopy (dSTORM) and stimulated emission depletion (STED) microscopy (Willig et al., 2006; Klein et al., 2014). Nanoscopy utilizes clever computational and optical strategies to surpass the diffraction limit of image resolution, approaching molecular scale discrimination of objects.

The articles submitted to this Research Topic fall into three overlapping categories: General reviews of optogenetics for the study of synaptic or circuit plasticity, methodological reports of novel experimental tools and algorithms using neurophotonic data, applications of optogenetics to understanding neurological disorders and basic brain circuit function.

A comprehensive and provocative review by Lee et al. covers the diverse optogenetic tools that have been applied to the art of “circuit-bashing” (i.e., functional and anatomical dissection of local and long-range connectivity) with a focus on experiments exploiting cell-type specific expression of optogenetic actuators and inhibitors. This article also discusses numerous important caveats and limitations of optogenetic methods. Other reviews in the Research Topic instead focus more sharply on applications of neurophotonics techniques in specific circuits, in particular the spinal cord (Harding et al.) and the visual system (Rangel Olguin et al.), including in depth discussions of calcium imaging and viral-based methods to study neuronal connectivity. Morales et al. examine memory encoding analyzed with optogenetic tools, while the use of optophysiology to reveal mechanisms underlying synaptic plasticity, and in particular the functional matching of pre- and postsynaptic efficacy, a question that lends itself particularly well to the subcellular resolution offered by optical approaches, is presented in a thought-provoking review by Sanderson et al.

One important objective of the CNP is the development and dissemination of useful optogenetic tools for neuroscience research. This Research Topic introduces a number of technological advances, ranging from CREMSCLE (Cre-Mediated Single Cell Labeling by Electroporation), a simple trick for labeling single neurons using easy-to-implement bulk electroporation methods (Schohl et al.) all the way to a full protocol for building and using an acousto-optic deflector-based random access 2-photon microscope together with software to facilitate high-frequency (3 Hz) functional imaging across hundreds of synaptic sites on a complex 3D dendritic arbor (Sakaki et al.).

The implantation of GRIN lenses and subsequent calcium signal acquisition via a head-mounted portable miniature microscope (a.k.a, “miniscope”) provides a powerful means for studying the brain of awake behaving animals engaged in more naturalistic tasks. While this powerful method holds great promise for understanding the relationship of behaviors to complex neuronal responses, like place cell activity in the deep hippocampus for example, one of the challenges of 1P calcium imaging *in vivo* is the proper segmentation of unfocused images as well as the conversion of fluorescence signals to electrophysiologically meaningful data. To this end, Tran et al. discuss the benefits of applying machine learning algorithms to single-cell region-of-interest identification. This work is complemented by an innovative approach developed by Etter et al. for predicting animal behaviors from neuronal calcium signals, surprisingly by entirely foregoing the temptation to try to infer spiking events and frequencies and instead using a naïve Bayesian classifier to generate a probability function to directly relate behaviors to complex calcium signals.

Despite the appeal of functional imaging from hundreds of neurons, sometimes the temporal resolution of electrode recording offers the best approach to understanding circuit function. Mocanu and Shmuel provide a detailed protocol and evaluation of the use of relatively low-resolution intrinsic signal optical imaging to target recording electrode penetrations or virus injection.

Original research reports presented in this Research Topic are similarly broad in scope, starting with two superresolution nanoscopy papers, one using STED and the other using dSTORM. The STED study cleverly applies machine learning approaches to generalize, based on measurements made across vast populations of synapses in culture, about the relative distances between specific pre and postsynaptic proteins under baseline conditions and following chemical induction of synaptic plasticity, thus providing novel insights into how the pre and postsynaptic terminals reorganize at the molecular level (Wiesner et al.). The dSTORM study instead examines the known phenomenon of cyclical pulsatile release of growth hormone (GH) driven by GH releasing hormone (GHRH) neurons in the hypothalamus (Bednarz et al.). Using superresolution imaging to identify excitatory and inhibitory synapses in GHRH neurons based on proximity of vglut2-PSD95 pairs and vgat-gephyrin pairs respectively, they made the remarkable finding that while inhibitory synapse number was relatively stable over the 3 h cycle of waxing and waning GH release, excitatory synapses dynamically formed and disassembled over the same period, presumably mediating the cyclical GH release.

Naturally, several papers based on optogenetic reagents for activation and inhibition of signaling featured as well. An innovative paper by Luyben et al. presents a new transgenic mouse line expressing a photoactivatable adenylyl cyclase (PAC) protein. Combining PAC activation with electrophysiology and voltage sensitive dye measurements, the authors demonstrated that a robust synaptic long-term potentiation in hippocampus could be induced by optogenetically elevating postsynaptic cAMP. The study by Lafferty and Britt offers more of a cautionary tale. They examined the consequences of optogenetic silencing of inputs to nucleus accumbens (NAc) using illumination of the light-driven proton pump ArchT on feeding behavior in mice. Remarkably, they found that fiber optic-mediated inhibition in the NAc of the axon terminals of basolateral amygdala and paraventricular thalamus inputs had dramatically different effects on feeding behavior than did direct somatic silencing of these input nuclei. The researchers provided a possible explanation for this difference by showing that the frequency of spontaneous excitatory postsynaptic currents onto NAc was unexpectedly elevated by axon terminal ArchT illumination, probably owing to local changes in pH. This meticulous study should serve as a valuable cautionary note to be aware of potential off-target effects whenever using optogenetic approaches.

Sitting at the other end of the imaging scale is an original research paper using mesoscopic imaging of the entire visual cortex in GCaMP6s transgenic mice (Laliberté et al.). The investigators sought to observe changes in stimulus selectivity over a week of presentation of conditioning stimuli favoring a specific stimulus orientation. They found that specifically under conditions of cholinergic enhancement, significant levels of stimulus conditioning took place. This series of studies from subsynaptic resolution molecular analysis of plasticity up to mesoscopic calcium imaging of the entire visual cortex reveal the impressive scope offered by neurophotonic approaches to explore plasticity mechanisms in the brain.

Finally, the most impactful promise of neurophotonics technology lies in its potential for helping us understand neurological disease and eventually to offer cures. For example, one of the very first proposals for the application of optogenetics was to treat blindness (Bi et al., 2006). Epilepsy is one of the most important neurological conditions for which basic neuroscience research has been able to yield invaluable pharmacological and surgical interventions. Animal models of seizure have been critical to advancing our understanding the etiology of this devastating condition. Neurophotonics can shed light here too. Cela and Sjöström report on their optogenetic kindling model which offers some of traditional hallmarks of electrical kindling models, but without the accompanying confound of physical damage and astrogliosis and with the possibility of cell-type specificity for a much deeper mechanistic understanding of the contributions of different cellular players in seizure.

In their forward-looking perspective article, Shen et al. discuss the promise and challenges of using optogenetics therapeutically to treat human neurological disease. In addition to its original application to treating blindness, the possibility of all-optical deep brain stimulation for treating movement disorders and optogenetic silencing as a way to target brain areas associated with devastating chronic pain are discussed, with a very pragmatic consideration of what improvements are needed for optogenetic constructs, such as red-shifting activation wavelengths to permit transcranial illumination and improving viral vectors for more targeted delivery with minimal antigenicity.

The rapidity and extent with which optogenetics has assumed a prominent place in basic and translational neuroscience research, going from a figurative to literal spark in someone's eye in just a few years, is awe inspiring and an explicit testament of its truly revolutionary nature. New reporters and activators are emerging constantly, with considerable excitement over the newest generation of genetically-encoded voltage indicators (Abdelfattah et al., 2019). With new opportunity also comes new challenges. Just finding efficient ways to store the vast quantities of data generated from long streams of volumetric fluorescence recordings, not to mention the increasing open science demands for sharing raw data online, poses a challenge that will require major international standardization initiatives, such as the Neurodata Without Borders project (Teeters et al., 2015). Fortunately, the neuroscience community has already proven itself eager to collaborate on these and other issues, and there is no shortage of innovators, as this Research Topic nicely confirms. The future of neurophotonics is bright.

## Author Contributions

The manuscript was written by ER and edited by J-CB and YD. All authors contributed to the article and approved the submitted version.

## Funding

This project was made possible by the Canadian Neurophotonics Platform (CNP), which has evolved into the Canadian Optogenetics and Vectorology Foundry, established in 2015 with generous funding from the Brain Canada Foundation, through the Canada Brain Research Fund, with the financial support of Health Canada and partners. The platform supports neuroscience research and training throughout Canada and internationally by promoting the development and dissemination of key reagents and tools for optogenetics, fiber photometry, and light microscopy-based research (RRID:SCR_016477).

## Conflict of Interest

The authors declare that the research was conducted in the absence of any commercial or financial relationships that could be construed as a potential conflict of interest.

## Publisher's Note

All claims expressed in this article are solely those of the authors and do not necessarily represent those of their affiliated organizations, or those of the publisher, the editors and the reviewers. Any product that may be evaluated in this article, or claim that may be made by its manufacturer, is not guaranteed or endorsed by the publisher.
